# Trauma blocking or resource buffering? Occupational stress, burnout, and existential anxiety in teachers bereaved during the COVID-19 pandemic: the domain-specific effect of work–family boundary flexibility

**DOI:** 10.3389/fpsyg.2026.1834810

**Published:** 2026-07-23

**Authors:** Xiang Ji, Yu Zuo, Yiya Xu, Peilai Jin, Cheng Lyu, Xiaodong Liu, Miaomiao Wang

**Affiliations:** 1 School of Teacher Education, Weifang University, Weifang, China; 2 Xinjiang Key Laboratory of Mental Development and Learning Science, Urumqi, China; 3 Guangxi College for Preschool Education, Nanning, China

**Keywords:** burnout, existential anxiety, occupational stress, pandemic-bereaved teachers, trauma blocking hypothesis, work–family boundary flexibility

## Abstract

**Background:**

The COVID-19 pandemic left deep psychological scars on teachers who lost loved ones. This population bore a dual burden—profound grief while fulfilling professional duties—yet their psychological processes remain understudied. Integrating conservation of resources theory with existential psychology, this study examined the relationship between occupational stress and existential anxiety in pandemic-bereaved teachers, the mediating role of burnout, and the differential functioning of four work–family boundary flexibility dimensions, proposing the trauma blocking hypothesis and reporting preliminary findings consistent with it.

**Method:**

A convenience sample of 1,103 secondary school teachers who lost immediate family members to COVID-19 completed measures of occupational stress, burnout, existential anxiety, and work–family boundary flexibility. Mediation and moderated mediation analyses were conducted using PROCESS, controlling for gender and age.

**Results:**

Occupational stress positively predicted existential anxiety. Burnout partially mediated this relationship (59.3% of total effect). The four flexibility dimensions dissociated: only work flexibility ability significantly moderated the stress–burnout pathway, amplifying (rather than buffering) the association. Work flexibility willingness, family flexibility ability, and family flexibility willingness showed no significant moderation.

**Conclusion:**

All four hypotheses received empirical support. Occupational stress translated into existential anxiety via resource depletion and meaning erosion embodied by burnout. Findings provide preliminary evidence for the trauma blocking hypothesis, suggesting domain-specific resource efficacy: family-related resources showed no moderation, whereas work flexibility ability shifted from buffering to amplifying. Because blocking mechanisms were not directly measured, interpretations need future support. Practically, educational administrators should prioritize objective work flexibility over encouraging bereaved teachers to “seek support from family.”

## Introduction

The COVID-19 pandemic, the most severe public health crisis of the 21st century, inflicted profound and enduring health and social consequences worldwide (World Health Organization, 2023). As societies gradually returned to a semblance of normalcy, the pandemic appeared to be receding from public memory. For those who lost loved ones during this period, however, psychological wounds remained unhealed. A survey of university teachers in Wuhan revealed that educators who lost family members to COVID-19 faced a 459% increased risk of post-traumatic stress disorder (PTSD) (Fan et al., 2021)—a stark demonstration of the devastating psychological impact of bereavement on this professional group.

This population bore a unique dual burden. Bereaved teachers confronted profound personal grief while simultaneously fulfilling their professional mandate to educate and provide emotional support to students at a critical developmental stage. Fraser (2022) documented that teachers experienced significant grief and burnout during the pandemic, with these pressures persisting long after the acute crisis subsided. Frei-Landau et al. (2024) reported that 70% of teachers had grieving students in their classrooms; yet research on how educators coped with their own grief remained conspicuously inadequate. Critically, the nature of their professional role often deprived bereaved teachers of the time and space needed to process their loss, rendering their suffering a form of disenfranchised grief—grief that was not publicly acknowledged or socially supported (Doka, 2002). This disenfranchisement rendered teachers’ psychological struggles highly invisible, leaving them systematically neglected in both academic inquiry and support provision.

For pandemic-bereaved teachers, the loss of a loved one triggered not merely emotional distress but a profound existential crisis. Yalom (1980) existential psychology identified four ultimate concerns confronting humanity: death, freedom, isolation, and meaninglessness. Bereavement directly confronted individuals with mortality, fundamentally destabilizing their beliefs about life’s meaning. Pascual-Soler et al. (2023) found that during the COVID-19 crisis, teachers exhibited higher levels of existential anxiety than students. Amirpour and Moradi (2022) further found that teachers’ existential thinking and sense of meaning in life negatively predicted their pandemic-related anxiety. Mahdavinoor et al. (2024) conceptualized existential anxiety as anxiety transcending objective threat and pertaining to existence itself, indicating its strong association with PTSD and negative correlations with self-esteem and emotion regulation. When existential anxiety was activated, psychological resources became rapidly depleted—a process that constituted the underlying mechanism of burnout. From an existential perspective, Pines (1993) argued that burnout originated from individuals’ fundamental need to believe their lives and work were meaningful. Loonstra et al. (2009) further showed that existential fulfilment played a significant role across burnout dimensions. For teachers already confronting meaning crises following bereavement, burnout may constitute a secondary assault on their sense of existential purpose, accelerating the deepening of existential anxiety. Accordingly, adopting a perspective that honored the lived experience of bereaved individuals, the present investigation selected existential anxiety as its focal outcome variable.

Occupational stress referred to the perceived imbalance between environmental demands and an individual’s coping capabilities. For pandemic-bereaved teachers, occupational stressors were multifaceted and cumulative: they navigated routine teaching tasks while simultaneously addressing students’ learning losses, fielding parental skepticism, and meeting heightened institutional expectations. Conservation of resources (COR) theory (Hobfoll, 1989, 2002) provided a robust framework for understanding how occupational stress undermined mental health, positing that individuals were intrinsically motivated to acquire, retain, and protect resources, and experienced stress when faced with actual or threatened resource loss. Bereavement itself constituted resource loss of the highest order: teachers lost emotional attachment figures and family support systems. When this fundamental resource depletion intersected with sustained occupational stress, educators confronted not only psychological resource exhaustion but also a fundamental questioning of life meaning—the ultimate personal resource. Yalom (1980) observed that confrontation with mortality inevitably triggered questioning of meaning. Thus, the prediction of existential anxiety by occupational stress represented the convergent operation of resource loss spirals and meaning erosion spirals. Amirpour and Moradi (2022) found that when individuals’ sense of meaning was threatened, their vulnerability to stress increased significantly. Pascual-Soler et al. (2023) found that teachers reported higher existential anxiety than students. Accordingly, Hypothesis 1 was proposed: Occupational stress positively predicted existential anxiety.

Maslach and Jackson (1981) conceptualized burnout as a three-dimensional construct comprising emotional exhaustion, depersonalization, and reduced personal accomplishment. Emotional exhaustion reflected resource depletion, while diminished personal accomplishment implicated threats to self-worth and meaning. Drawing on COR theory, De Cuyper et al. (2012) described resource loss spirals, whereby reduced resource perception exacerbated exhaustion, which further undermined resource perception in a self-perpetuating cycle. Teachers constituted a population at elevated risk for burnout (Watlington et al., 2010), bearing the dual demands of instructional and emotional labor. For bereaved teachers, diminished personal accomplishment directly activated profound questioning of work’s value and meaning. Pines (1993) located the fons et origo of burnout in individuals’ need to believe their lives and work were meaningful. While Loonstra et al. (2009) found that existential fulfilment operated significantly across burnout dimensions. Bereavement had already destabilized teachers’ fundamental beliefs about life’s meaning; burnout—particularly reduced personal accomplishment—may intensify existential anxiety. Occupational stress, through resource depletion and meaning erosion, translated into deeper existential crisis, with burnout serving as the mediating mechanism. Hypothesis 2 was therefore proposed: Burnout mediated the relationship between occupational stress and existential anxiety.

Identifying factors that might buffer the transformation of occupational stress into existential anxiety via burnout constituted a critical research priority. Work–family border theory (Clark, 2000) posited that individuals’ capacity to control boundaries between work and family domains determined their effectiveness in managing demands from both spheres. Boundary flexibility referred to the perceived latitude to alter when, where, and how domain responsibilities were fulfilled. Blazhevska Stoilkovska (2021) validated a four-dimensional structure of boundary flexibility in teacher samples: (1) work flexibility ability (objective capacity to adjust work arrangements), (2) work flexibility willingness (subjective motivation to utilize flexible arrangements), (3) family flexibility ability (objective conditions enabling flexible family arrangements), and (4) family flexibility willingness (subjective motivation to engage in family matters). In normative occupational contexts, these four dimensions typically functioned as integrated resources. Whether this structure dissociated in the context of bereavement—specifically, whether family-related dimensions became disabled by trauma—represented a critical unanswered question requiring systematic investigation.

Core mechanisms from trauma psychology provided theoretical grounding for examining this dissociation. First, the cognitive-behavioral model of PTSD (Ehlers and Clark, 2000) established that traumatized individuals developed avoidance patterns in response to trauma-related cues. Second, attachment theory (Mikulincer and Shaver, 2007) indicated that trauma activated insecure attachment patterns, undermining individuals’ capacity to derive security from close relationships. Integrating these perspectives yielded a compelling theoretical proposition: the family constituted not merely the physical setting where trauma occurred but also the symbolic locus of secure attachment. Bereavement entailed the permanent loss of an attachment figure, psychologically rupturing the family as a previously safe base. When teachers attempted to mobilize family flexibility resources, trauma-related avoidance mechanisms were inevitably triggered; simultaneously, insecure attachment patterns were activated. This dual disruption rendered family-related resource dimensions psychologically inaccessible and functionally inoperative. In marked contrast, work flexibility ability represented a structural resource relatively dissociated from the trauma source—it required no emotional investment, triggered no traumatic memories, and provided objective control over work parameters. Monn et al. (2018) showed that inhibitory control significantly moderated the relationship between trauma exposure and PTSD symptoms. Integrating border theory with trauma psychology, Hypothesis 3 was proposed: Work flexibility ability moderated the occupational stress–burnout pathway, such that higher work flexibility ability attenuated the positive association between stress and burnout. Hypothesis 4 posited that family flexibility ability, family flexibility willingness, and work flexibility willingness would show no significant moderating effects, which would be consistent with the possibility that trauma-related processes may impede these resources. However, non-significant findings do not constitute direct evidence of such impediment, and alternative explanations remain possible.

In summary, the present investigation addressed three significant gaps in the literature. First, the occupational psychological processes of pandemic-bereaved teachers remained critically understudied (Fraser, 2022; Frei-Landau et al., 2024), with the psychological mechanisms underlying their dual trauma–occupational burden likely diverging fundamentally from those operating in non-bereaved educators. Second, the internal differentiation of work–family boundary flexibility under traumatic conditions had not been systematically examined (Clark, 2000; Blazhevska Stoilkovska, 2021); the present study introduced the trauma blocking hypothesis, proposing that family-related dimensions may become inoperative due to avoidance and emotional numbing. Third, existential anxiety—a core psychological experience for bereaved populations—had been conspicuously neglected in occupational psychology (Yalom, 1980; Neimeyer, 2001). By testing these hypotheses, the present investigation aimed to make three theoretical contributions: directing scholarly attention to a forgotten population of bereaved teachers; deconstructing the boundary flexibility construct contextually to propose domain-specific resource efficacy in traumatic contexts; and integrating conservation of resources theory with existential psychology to construct a comprehensive explanatory framework linking external occupational pressure to internal meaning crisis.

## Methods

### Participants and procedure

The study focused on secondary school teachers who lost loved ones during the COVID-19 pandemic. A convenience sampling method was employed to recruit participants from Wuhan and surrounding areas in Hubei Province between March and June 2025. Inclusion criteria were: (1) currently employed as a secondary school teacher (including junior and senior high school); (2) loss of an immediate family member (parent, spouse, or child) due to COVID-19 during the 2020–2022 pandemic period; and (3) voluntary participation. Exclusion criteria were: (1) incomplete questionnaire completion (missing rate > 10%); (2) overtly patterned responding; and (3) classification as invalid by two independent researchers.

Approval for the study was obtained from the Research Ethics Committee of Weifang University. Given the vulnerable nature of the participant population, all procedures strictly adhered to trauma-informed care principles. Prior to data collection, researchers provided potential participants with detailed information regarding the study purpose, procedures, confidentiality protections, and voluntary participation rights; written informed consent was subsequently obtained. Questionnaires were completed anonymously in quiet, private meeting rooms, with researchers present throughout to provide support. Completion time was approximately 25–30 min, after which questionnaires were sealed and collected immediately on site. Upon conclusion of data collection, all participants were provided with information about mental health resources for future reference should they require assistance.

A total of 1,170 questionnaires were distributed, and 1,103 valid responses were retrieved, yielding an effective response rate of 94.27%. The sample comprised 839 female teachers (76.1%) and 264 male teachers (23.9%). Age distribution was as follows: 20–30 years, 194 (17.6%); 31–40 years, 236 (21.4%); 41–50 years, 453 (41.1%); 51–60 years, 220 (19.9%). Teaching experience was distributed as: ≤5 years, 226 (20.5%); 5–10 years, 129 (11.7%); 11–20 years, 103 (9.3%); 21–30 years, 466 (42.2%); >30 years, 179 (16.2%). Bereaved family members included parents (*n* = 473, 42.9%), spouses (*n* = 533, 48.3%), and children (*n* = 97, 8.8%).

## Measures

### Occupational stress

Occupational stress was assessed using the Occupational Stress Questionnaire for Primary and Secondary School Teachers, developed by Zhu (2003). The scale comprised 46 items across six dimensions: examination pressure (8 items), student factors (12 items), self-development needs (9 items), interpersonal relationships (7 items), workload (6 items), and career expectations (4 items). Items were rated on a 5-point Likert scale, with higher total scores indicating greater occupational stress. In the present study, Cronbach’s *α* for the total scale was 0.937, with subscale *α* coefficients ranging from 0.687 to 0.837, indicating good reliability.

### Burnout

Burnout was measured using the revised version of the Teacher Burnout Questionnaire for Primary and Secondary School Teachers (Zheng, 2013). This 17-item instrument comprised three dimensions: emotional exhaustion (5 items), depersonalization (6 items), and reduced personal accomplishment (6 items). Items were rated on a 5-point Likert scale, with items 4, 5, 6, 7, 8, and 10 reverse-scored. Higher total scores indicated more severe burnout. In the present study, Cronbach’s *α* for the total scale was 0.896, with subscale *α* coefficients ranging from 0.831 to 0.899.

### Existential anxiety

Existential anxiety was measured using the Existential Anxiety Scale developed by Chen and Wang (2010). The 27-item scale comprised four dimensions: death anxiety and fate anxiety (8 items), meaninglessness anxiety and emptiness anxiety (6 items), condemnation anxiety and guilt anxiety (7 items), and alienation anxiety and loneliness anxiety (6 items). Items were rated on a 5-point Likert scale, with items 3, 5, 7, 8, 9, 10, 12, 13, 22, and 27 reverse-scored. Higher total scores indicated higher levels of existential anxiety. In the present study, Cronbach’s *α* for the total scale was 0.870. Because two subscales—meaninglessness anxiety and emptiness anxiety (0.519) and condemnation anxiety and guilt anxiety (0.449)—demonstrated internal consistency below acceptable levels, subsequent analyses employed only the two subscales with satisfactory reliability: death anxiety and fate anxiety (0.778) and alienation anxiety and loneliness anxiety (0.724). No further analyses used the total scale score or the two unreliable subscales.

### Work–family boundary flexibility

Work-family boundary flexibility was assessed using the Work-Family Boundary Flexibility Scale developed by Matthews et al. (2010) and validated in teacher samples by Blazhevska Stoilkovska (2021). The 16-item instrument comprised four dimensions: work flexibility ability (4 items), family flexibility ability (4 items), work flexibility willingness (5 items), and family flexibility willingness (3 items). Items were rated on a 5-point Likert scale, with no reverse-scored items. Each dimension was scored separately, with higher scores indicating greater flexibility in that domain. In the present study, Cronbach’s *α* for the total scale was 0.761, with subscale *α* coefficients ranging from 0.734 to 0.900. Although this scale has not been formally validated in a Chinese population, its internal consistency in the present study was satisfactory (Cronbach’s α ranging from 0.734 to 0.900 for the four dimensions). To further assess its construct validity, a confirmatory factor analysis (CFA) was conducted using Mplus 8.0 with robust maximum likelihood estimation. The four-factor model (work flexibility ability, work flexibility willingness, family flexibility ability, family flexibility willingness) showed acceptable fit to the data: χ^2^/df = 2.98, CFI = 0.912, RMSEA = 0.068, SRMR = 0.055. All factor loadings were significant (*p* < 0.001), ranging from 0.61 to 0.89. These results support the four-dimensional structure originally reported by Matthews et al. (2010) and Blazhevska Stoilkovska (2021) in the Chinese teacher sample. Future research should formally validate the scale in broader Chinese populations.

### Data analysis strategy

Data were analyzed using SPSS 26.0 and the PROCESS macro (Hayes, 2022).

*Preliminary analyses*: Means, standard deviations, and Pearson correlation coefficients were computed for all study variables. Harman’s single-factor test was conducted to assess common method bias.

*Mediation analysis*: PROCESS Model 4 was employed to test the mediating role of burnout in the relationship between occupational stress and existential anxiety (Hypothesis 2). Bias-corrected bootstrapping with 5,000 resamples was used to generate 95% confidence intervals for indirect effects. Mediation was considered significant if the confidence interval excluded zero.

*Moderated mediation analysis*: PROCESS Model 7 was used to test the moderating effects of the four work–family boundary flexibility dimensions (work flexibility ability, work flexibility willingness, family flexibility ability, family flexibility willingness) on the occupational stress–burnout pathway (Hypotheses 3 and 4). For each dimension, the following were examined: (1) the significance of the interaction term between the focal dimension and occupational stress; (2) conditional indirect effects at different levels of the moderator (*M* ± 1*SD*); and (3) the index of moderated mediation. Moderation was considered significant if the interaction term was significant and the conditional indirect effects confidence intervals excluded zero.

All analyses controlled for demographic variables (gender, age, years of teaching experience, and time since bereavement). Statistical significance was set at *α* = 0.05.

## Results

### Common method bias

Harman’s single-factor test was conducted on all self-report items using unrotated exploratory factor analysis. The results revealed 19 factors with eigenvalues greater than 1, with the first factor accounting for 21.04% of the total variance, below the threshold of 40%. These findings indicated that common method bias was not a serious concern in the present study.

### Descriptive statistics and correlational analyses

Means, standard deviations, and Pearson correlation coefficients for all study variables are presented in Table 1. The correlational analyses showed the following:

**Table 1:** Means, standard deviations, and correlations among study variables (*N* = 1,103).

Variable	*M*	*SD*	1	2	3	4	5
1 Gender	1.76	0.427					
2 Age	2.63	0.992	−0.272**				
3 OCS	3.04	0.526	−0.152**	0.195**			
4 BOT	2.24	0.538	−0.112**	−0.007	0.556**		
5 WFB	3.08	0.432	−0.045	0.174**	0.185**	0.141**	
6 EXA	2.34	0.542	−0.097**	−0.015	0.555**	0.718**	0.141**

Gender coding: 1 = male, 2 = female. OCS, Occupational Stress; BOT, Burnout; EXA, Existential Anxiety; WFB, Work–Family Boundary Flexibility. *p < 0.05, **p < 0.01.

Occupational stress was significantly positively correlated with existential anxiety (*r* = 0.555, *p* < 0.01), providing preliminary support for Hypothesis 1. Occupational stress was significantly positively correlated with burnout (*r* = 0.556, *p* < 0.01), and burnout was significantly positively correlated with existential anxiety (*r* = 0.718, *p* < 0.01), satisfying the prerequisites for testing mediation.

Notably, work–family boundary flexibility was significantly positively correlated with occupational stress (*r* = 0.185, *p* < 0.01), burnout (*r* = 0.141, *p* < 0.01), and existential anxiety (*r* = 0.141, *p* < 0.01). These findings indicated that, within this study population, boundary flexibility did not function as a simple protective factor; higher flexibility was associated with higher levels of stress, burnout, and anxiety. This ostensibly counterintuitive pattern suggested that the mechanism of boundary flexibility may operate differently in pandemic-bereaved teachers compared to normative populations, providing important preliminary evidence for subsequent moderation analyses.

Significant correlations were also observed between demographic and core variables. Gender was negatively correlated with occupational stress (*r* = −0.152, *p* < 0.01), burnout (*r* = −0.112, *p* < 0.01), and existential anxiety (*r* = −0.097, *p* < 0.01), indicating that female teachers reported higher levels of stress, burnout, and anxiety. Age was positively correlated with occupational stress (*r* = 0.195, *p* < 0.01) but not significantly correlated with burnout (*r* = −0.007, *p* > 0.05) or existential anxiety (*r* = −0.015, *p* > 0.05). Accordingly, gender and age were included as control variables in subsequent analyses.

Correlations between additional demographic variables (years of teaching experience, time since bereavement, and type of bereaved family member) and the core study variables were also examined. Years of teaching experience showed a small negative correlation with burnout (*r* = −0.09, *p* < 0.01) but no significant correlation with occupational stress or existential anxiety. Time since bereavement (in months) was not significantly correlated with any core variable (all *r* < 0.05, *p* > 0.10). Type of bereaved family member (dummy-coded as spouse vs. parent vs. child) did not show significant main effects on any core variable when entered as a covariate. These results suggest that the observed relationships are not substantially confounded by these demographic factors.

### Mediation analysis

PROCESS macro Model 4 (Hayes, 2022) was employed to test the mediating role of burnout in the relationship between occupational stress and existential anxiety (Hypothesis 2). Bias-corrected bootstrapping with 5,000 resamples was used to generate 95% confidence intervals for indirect effects.

As shown in Table 2, the total effect of occupational stress on existential anxiety was significant (*B* = 0.174, *SE* = 0.008, *t* = 22.61, *p* < 0.001, 95% CI [0.159, 0.189]). After controlling for the mediator, the direct effect of occupational stress on existential anxiety remained significant (*B* = 0.071, *SE* = 0.008, *t* = 9.29, *p* < 0.001, 95% CI [0.056, 0.086]).

**Table 2:** Mediation analysis of burnout.

Effect	Estimate	Boot *SE*	*t*	*p*	95%CI	Prop.
OCS → BOT (a)	0.210	0.010	22.22	< 0.01	[0.192, 0.229]	
BOT → EXA (b)	0.491	0.020	24.32	< 0.01	[0.452, 0.531]	
OCS → EXA (c’)	0.071	0.008	9.29	< 0.01	[0.056, 0.086]	40.7%
Total effect (c)	0.174	0.008	22.61	< 0.01	[0.159, 0.189]	
Indirect effect (a × b)	0.103	0.007			[0.090, 0.117]	59.3%

*N* = 1,103. Bootstrap resamples = 5,000. CI, confidence interval. Total effect is the sum of direct and indirect effects. Proportion is based on total effect.

Path analysis indicated that occupational stress significantly and positively predicted burnout (*a* = 0.210, *SE* = 0.010, *t* = 22.22, *p* < 0.001, 95% CI [0.192, 0.229]), and burnout significantly and positively predicted existential anxiety (*b* = 0.491, *SE* = 0.020, *t* = 24.32, *p* < 0.001, 95% CI [0.452, 0.531]).

Mediation analysis indicated that the indirect effect of occupational stress on existential anxiety through burnout was 0.103 (Boot *SE* = 0.007), with a bootstrap 95% confidence interval of [0.0904, 0.1166] excluding zero, confirming a significant mediating effect. The indirect effect accounted for 59.3% of the total effect. Thus, Hypothesis 2 was supported: burnout partially mediated the relationship between occupational stress and existential anxiety.

### Moderated mediation analysis

PROCESS macro Model 7 (Hayes, 2022) was used to test the moderating effects of the four work–family boundary flexibility dimensions (work flexibility ability, work flexibility willingness, family flexibility ability, family flexibility willingness) on the occupational stress–burnout pathway (Hypotheses 3 and 4). Bias-corrected bootstrapping with 5,000 resamples was employed to generate conditional indirect effects and the index of moderated mediation. Results are presented in Table 3.

**Table 3:** Moderated mediation analysis for the four boundary flexibility dimensions.

Moderator (M)	*B*	*SE*	*p*	95% CI	Effect(Boot 95% CI)	Index(Boot 95% CI)
WFA	0.0060	0.0026	0.020	[0.0009, 0.0110]	−1SD: 0.0868 [0.0728, 0.1017] Mean: 0.0955 [0.0833, 0.1078] +1SD: 0.1043 [0.0892, 0.1200]	0.0029 [0.0001, 0.0057]
WFW	0.0022	0.0024	0.344	[−0.0024, 0.0069]	−1SD:0.0863 [0.0719, 0.1015] Mean:0.0896 [0.0774, 0.1028] +1SD: 0.0929 [0.0770, 0.1100]	0.0011 [−0.0019, 0.0043]
FFA	−0.0004	0.0022	0.859	[−0.0048, 0.0040]	−1SD:0.1011 [0.0868, 0.1167] Mean:0.1003 [0.0878, 0.1138] +1SD: 0.0997 [0.0836, 0.1165]	−0.0002 [−0.0029, 0.0023]
FFW	−0.0037	0.0033	0.259	[−0.0102, 0.0028]	−1SD:0.1058 [0.0907, 0.1224] Mean: 0.1003 [0.0874, 0.1137] +1SD: 0.0985 [0.0837, 0.1133]	−0.0018 [−0.0061, 0.0021]

*N* = 1,103. Bootstrap = 5,000 resamples. Conditional indirect effects are reported at three levels of the moderator: low (−1 SD), mean, and high (+1 SD), corresponding to the 16th, 50th, and 84th percentiles, respectively. The significance of the index of moderated mediation is indicated by a 95% bootstrap confidence interval that does not include zero. Bold indicates a statistically significant moderating effect. WFA, work flexibility ability, WFW, work flexibility willingness, FFA, family flexibility ability, FFW, family flexibility willingness.

Moderation analyses showed a clear dissociation among the four dimensions:

*Work flexibility ability (WFA)*: The interaction term between occupational stress and work flexibility ability significantly predicted burnout (*B* = 0.0060, *SE* = 0.0026, *t* = 2.33, *p* = 0.020, 95% CI [0.0009, 0.0110]), indicating that work flexibility ability significantly moderated the occupational stress–burnout pathway.
*Work flexibility willingness (WFW)*: The interaction term was not significant (*B* = 0.0022, *SE* = 0.0024, *p* = 0.344).
*Family flexibility ability (FFA)*: The interaction term was not significant (*B* = −0.0004, *SE* = 0.0022, *p* = 0.859).
*Family flexibility willingness (FFW)*: The interaction term was not significant (*B* = −0.0037, *SE* = 0.0033, *p* = 0.259).

Conditional indirect effect analysis demonstrated that at low (6), medium (9), and high (12) levels of work flexibility ability, the indirect effects of occupational stress on existential anxiety through burnout were 0.0868 (Boot CI [0.0728, 0.1017]), 0.0955 (Boot CI [0.0833, 0.1078]), and 0.1043 (Boot CI [0.0892, 0.1200]), respectively. As work flexibility ability increased, the indirect effect progressively strengthened. The index of moderated mediation was 0.0029 (Boot *SE* = 0.0014, 95% Boot CI [0.0001, 0.0057]), with the confidence interval excluding zero, providing further evidence for the significant moderating effect of work flexibility ability. The indices of moderated mediation for the remaining three dimensions all had confidence intervals containing zero, indicating that their moderating effects were not significant.

The results showed a differentiated pattern of the four work–family boundary flexibility dimensions in the traumatic context. Only work flexibility ability—a dimension directly related to the work domain and reflecting structural, objective capacity—significantly moderated the transformation of occupational stress into burnout. Specifically, as work flexibility ability increased, the effect of occupational stress on burnout became stronger, indicating an amplifying rather than buffering moderating pattern. These findings supported Hypothesis 3, confirming that work flexibility ability moderated the first stage of the mediated pathway.

More importantly, consistent with the statistical prediction of Hypothesis 4, work flexibility willingness, family flexibility ability, and family flexibility willingness demonstrated no significant moderating effects. This dissociation was consistent with the trauma blocking hypothesis. However, because non-significant results do not prove the absence of effects, caution is advised against interpreting this pattern as direct evidence of trauma-induced blocking. Alternative explanations—such as measurement limitations or insufficient variance in these dimensions—cannot be ruled out. The finding that work-related structural resources remained operative, while family-related dimensions did not show significant moderation, is suggestive but requires direct testing of mediating mechanisms (e.g., avoidance, emotional numbing) in future research.

**Figure 1:**
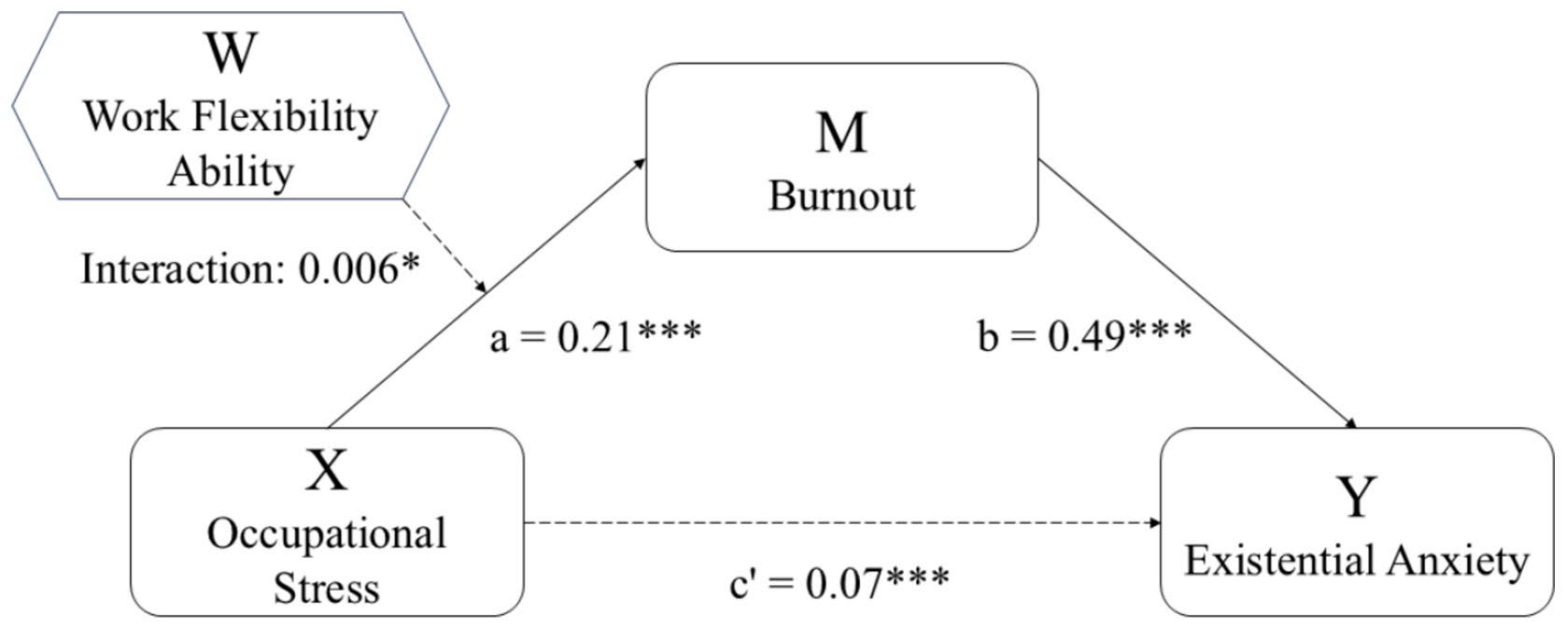
Path diagram of the moderated mediation model. Values are unstandardized coefficients. OCS, Occupational Stress; BOT, Burnout; EXA, Existential Anxiety; WFA, Work Flexibility Ability. The solid arrows represent direct and indirect paths; the dashed arrow represents the direct effect of OCS on EXA after controlling for the mediator. The interaction term (OCS × WFA) predicts BOT, indicating that WFA moderates the first stage of the mediation pathway. Control variables (gender, age, teaching experience, time since bereavement) are not shown for simplicity. *p < 0.05, **p < 0.01, ***p < 0.001.

The path diagram of the moderated mediation model is displayed in Figure 1.

### Robustness checks

To examine the robustness of the findings, the demographic variables of gender, age, and years of teaching experience were included as control variables in the moderated mediation model. The pattern of results remained unchanged. The indirect effect of occupational stress on existential anxiety through burnout remained significant (indirect effect = 0.1045, Boot *SE* = 0.0070, 95% CI [0.0910, 0.1185]). Work flexibility ability (WFA) continued to significantly moderate the occupational stress–burnout pathway (interaction *B* = 0.0061, *SE* = 0.0025, *p* = 0.0175, 95% CI [0.0011, 0.0111]), with an index of moderated mediation of 0.0029 (Boot *SE* = 0.0014, 95% Boot CI [0.0002, 0.0056]). Work flexibility willingness (WFW), family flexibility ability (FFA), and family flexibility willingness (FFW) remained non-significant moderators (interaction *p* values = 0.2906, 0.8833, and 0.2618, respectively; all 95% bootstrap confidence intervals for the indices of moderated mediation contained zero). These results indicated that the core findings of the study were robust and not dependent on the selection of specific control variables.

## Discussion

The present findings indicated that occupational stress significantly and positively predicted existential anxiety among pandemic-bereaved teachers (Hypothesis 1), a relationship that remained robust after controlling for demographic variables. This result aligned closely with the core tenets of conservation of resources (COR) theory (Hobfoll, 2002), which posited that individuals experienced stress when confronted with actual or threatened resource loss, and that such stress could precipitate deeper psychological crises. For pandemic-bereaved teachers, bereavement itself constituted resource loss of the highest order—encompassing the loss of emotional attachment figures and family support systems. Within this context of profound resource depletion, sustained occupational stress functioned as an ongoing resource threat, intensifying teachers’ existential concerns regarding life’s meaning, mortality, freedom, and isolation. The present findings engaged in substantive dialogue with recent empirical work on teacher existential anxiety. Pascual-Soler et al. (2023), studying a Spanish teacher sample, found that educators exhibited higher levels of existential anxiety than students during the COVID-19 crisis, with work pressure emerging as a significant predictor. Mahdavinoor et al. (2024), investigating an Iranian population, found strong associations between existential anxiety and mental health difficulties, identifying workload as a key correlate. The present study extended these findings by showing that, within a trauma-exposed population of bereaved teachers, the effect of occupational stress on existential anxiety was particularly pronounced—bereavement experiences amplified the capacity of occupational stress to trigger existential crisis. Notably, Amirpour and Moradi (2022) found that teachers’ existential thinking and sense of meaning in life negatively predicted their pandemic-related anxiety. Integrating these findings with the present results suggested that occupational stress among bereaved teachers may not only directly trigger existential anxiety but also indirectly exacerbate existential crisis through the erosion of meaning. This inference received support from Schnell and Krampe (2020), who showed that meaning mediated the relationship between stress and mental health, with this effect being particularly pronounced in trauma-exposed populations. In summary, the confirmation of Hypothesis 1 highlighted the distinctive nature of bereaved teachers’ psychological crisis: for educators who had lost loved ones, sustained occupational pressure transcended mere emotional distress, constituting a fundamental questioning of life’s meaning—a secondary assault on their sense of existential purpose.

The results indicated that burnout significantly mediated the relationship between occupational stress and existential anxiety (Hypothesis 2), providing a critical mechanistic explanation for how occupational stress translated into existential crisis. As teachers’ emotional resources became depleted and their sense of personal accomplishment diminished, deeper questioning of work’s value and life’s meaning was activated, thereby intensifying existential anxiety. These findings resonated with Loonstra et al.' (2009) classic investigation, which found significant negative correlations between existential fulfilment and all burnout dimensions in a sample of 504 Dutch secondary school teachers. Loonstra and colleagues proposed existential fulfilment as a vital psychological resource protecting teachers against burnout. The present study extended this work by illuminating the reverse pathway: burnout not only resulted from existential factors but also served as an antecedent to existential anxiety, creating a self-perpetuating cycle. For example, Schnell and Krampe (2020) found that meaning in life mediated the relationship between stress and mental health, with this effect being particularly pronounced in trauma-exposed populations. Arslan and Allen (2021) further showed that psychological flexibility and meaning in life operated as serial mediators in the relationship between coronavirus stress and subjective well-being among teachers. Arslan and Allen (2021), surveying Turkish participants, found that psychological flexibility and meaning in life operated as serial mediators in the relationship between coronavirus stress and subjective well-being. For bereaved teachers, this mediating mechanism carried particular significance: the death of loved ones had already destabilized fundamental beliefs about life’s meaning, and burnout—particularly reduced personal accomplishment—further amplified self-questioning regarding whether one’s work retained significance. In conclusion, the confirmation of Hypothesis 2 suggested a transformative pathway from external pressure to internal meaning crisis: occupational stress, through the dual pathway of resource depletion and meaning erosion embodied by burnout, ultimately crystallized as existential anxiety.

The results partially supported Hypothesis 3, indicating that work flexibility ability significantly moderated the occupational stress–burnout pathway, though the direction of moderation contradicted initial expectations. Contrary to the hypothesized buffering effect, higher work flexibility ability intensified, rather than attenuated, the positive association between occupational stress and burnout. According to COR theory, resources did not invariably function as buffers; under certain conditions, they may paradoxically become amplifiers—a phenomenon Hobfoll (2002) termed the resource investment paradox. Brotheridge and Lee (2002), in their investigation of emotional labor within a COR framework, found that when job demands were excessive, resource investment could accelerate rather than alleviate depletion. For bereaved teachers, work flexibility ability provided objective latitude to adjust work parameters, yet this very flexibility may entail a “nowhere to hide” dynamic—the capacity to work anytime, anywhere potentially blurred boundaries between work and recovery, increasing rather than decreasing psychological resource encroachment. An existential perspective revealed a deeper psychological mechanism: when individuals were immersed in the existential anxiety activated by bereavement, excessive work investment may function as an unconscious defense mechanism against confronting mortality and meaninglessness. This “busyness as anesthesia” strategy, paradoxically, accelerated psychological resource depletion. The resource-amplifying rather than buffering pattern found corroboration in recent research. Prior research has suggested that a U-shaped relationship between work flexibility and burnout, with moderate flexibility proving beneficial but excessive flexibility exacerbating exhaustion—a phenomenon they termed the flexibility paradox. Monzani and colleagues’ findings suggested the possibility of nonlinear relationships between work flexibility ability and burnout. For already resource-depleted bereaved teachers, work flexibility ability may represent a double-edged sword: while providing a sense of control, it simultaneously blurred the boundaries necessary for psychological recovery. In summary, the confirmation of Hypothesis 3 highlighted the complexity of resource functioning in traumatic contexts: identical resources may serve fundamentally different functions across varying contexts and populations, suggesting that future research should prioritize contextual resource efficacy over mere resource quantity.

The results showed that work flexibility willingness, family flexibility ability, and family flexibility willingness exhibited no significant moderating effects, a pattern consistent with the statistical expectation of Hypothesis 4. This dissociation was suggestive of the trauma blocking hypothesis, but several important caveats must be emphasized. First, non-significant findings do not constitute evidence that these resources are ‘blocked’ or ‘ineffective’; they merely indicate that no statistically significant moderation was detected under the conditions of the present study. Second, the proposed mechanisms underlying the trauma blocking hypothesis—avoidance, emotional numbing, and insecure attachment—were not directly measured. Therefore, any interpretation invoking these mechanisms remains speculative.

With these caveats in mind, a tentative explanation is offered that may guide future research. Integrating core mechanisms from trauma psychology, one could hypothesize that for bereaved teachers, the family constitutes the core setting where trauma occurred; family-related concepts and situations might trigger traumatic memories, potentially leading to avoidance. This could, in theory, prevent family flexibility resources from being mobilized effectively. Similarly, post-traumatic emotional numbing might undermine the translation of work flexibility willingness into actual behaviors. However, these are hypotheses to be tested, not conclusions drawn from the present data. Nevertheless, future research should directly measure PTSD symptoms, avoidance behaviors, attachment insecurity, and emotional numbing to rigorously test whether these mechanisms mediate the observed dissociation.

### Contributions and implications

The present study offered four principal contributions. First, it identified the distinctive psychological mechanisms operating in a previously neglected population—pandemic-bereaved teachers—responding directly to Fraser’s (2022) and Frei-Landau et al. (2024) calls for research addressing this dual-burden group. Second, it proposed the trauma blocking hypothesis and provided preliminary empirical findings consistent with it, suggesting that trauma may serve as a critical boundary condition for boundary theory. By documenting, for the first time, a clear dissociation among the four dimensions of work–family boundary flexibility in a traumatic context, this investigation extended the applicability of work–family border theory and enriched COR theory by directing attention toward the question of resource efficacy under trauma. However, because the proposed blocking mechanisms were not directly measured, the hypothesis awaits direct empirical testing. Third, it constructed a comprehensive explanatory framework linking external pressure to internal meaning crisis, integrating COR theory’s resource loss spirals with existential psychology’s meaning crisis conceptualization. This integrated framework offered novel theoretical perspectives for understanding occupational mental health in traumatized populations while providing empirical foundations for targeted intervention. Fourth, the findings provided clear practical guidance for educational administrators and institutions: prioritizing objective work flexibility arrangements (e.g., flexible scheduling, task autonomy) over encouraging bereaved teachers to “seek support from family,” while simultaneously monitoring teachers’ burnout and existential anxiety levels to facilitate meaning-centered interventions supporting post-bereavement meaning reconstruction.

### Limitations and future directions

Several limitations warrant consideration. First, like all cross-sectional studies, causal inferences cannot be drawn from the present data, and the sample was geographically limited to Wuhan and surrounding areas, which may restrict generalizability.

More importantly, several study-specific limitations should be noted.

Second, two subscales of the existential anxiety measure demonstrated suboptimal reliability (*α* = 0.519 and 0.449). This may reflect the unique characteristics of the participant population—bereavement experiences may fundamentally alter how individuals experienced and interpreted meaninglessness and guilt-related anxiety compared to normative populations. Future research should develop existential anxiety measures specifically validated for Chinese teacher populations, particularly those with trauma exposure, with Mahdavinoor et al.’ (2024) ECQ validation study providing methodological guidance.

Third, the study did not examine potential moderating or mediating effects of variables such as time since bereavement, social support, or meaning reconstruction processes. Future research should investigate these factors’ influences on core relationships to achieve more nuanced understanding of this population’s heterogeneity and the dynamic processes underlying psychological adaptation.

Fourth, the present study did not directly measure the psychological mechanisms hypothesized to underlie the trauma blocking effect—namely, avoidance of trauma-related cues, post-traumatic emotional numbing, or insecure attachment. Consequently, the interpretation that family-related resources were ‘blocked’ due to these processes remains speculative. Future research should include validated measures of PTSD symptoms (e.g., PCL-5), avoidance behaviors, and attachment styles to test the hypothesized mediating pathways directly. Without such direct evidence, the trauma blocking hypothesis should be regarded as a tentative framework requiring further empirical scrutiny.

Fifth, the present study did not statistically control for the type of bereaved family member (parent vs. spouse vs. child) due to imbalanced subgroup sizes (spouse: 48.3%, parent: 42.9%, child: 8.8%), which would reduce statistical power for interaction analyses. Theoretically, the focus was on the shared trauma of bereavement rather than differential effects of specific relationships. Nevertheless, post-hoc exploratory analyses were conducted entering bereaved family member type as a dummy-coded covariate; the core results (occupational stress → burnout → existential anxiety; work flexibility ability as moderator) remained unchanged in direction and significance. Future studies with larger and more balanced samples should examine whether the type of loss moderates the observed relationships. Additionally, participants’ attachment styles were not measured (e.g., secure vs. insecure attachment), which may influence resource mobilization after bereavement. Future research should include validated attachment measures to test this possibility.

## Conclusion

All four hypotheses proposed in this investigation received empirical support from the present data, with the caveats noted below.

First, occupational stress significantly and positively predicted existential anxiety among pandemic-bereaved teachers (Hypothesis 1 supported). This finding was consistent with the integrated framework of conservation of resources theory and existential psychology: bereavement, as resource loss of the highest order, may fundamentally destabilize teachers’ beliefs about life’s meaning, while sustained occupational pressure may transform routine workload demands into existential preoccupations concerning death, freedom, isolation, and meaninglessness.

Second, burnout partially mediated the relationship between occupational stress and existential anxiety (Hypothesis 2 supported), accounting for 59.3% of the total effect. This result suggested a transformative pathway from external pressure to internal meaning crisis: occupational stress, through the dual mechanism of resource depletion and meaning erosion embodied by burnout—specifically, the exhaustion of emotional resources and the diminishment of personal accomplishment—may ultimately crystallize as existential anxiety.

Third, work flexibility ability significantly moderated the occupational stress–burnout pathway (Hypothesis 3 supported), though the direction of moderation contradicted initial expectations. Contrary to a buffering effect, higher work flexibility ability intensified the positive association between occupational stress and burnout. This finding indicated the complexity of resource functioning in traumatic contexts: work flexibility ability, as a structural resource relatively dissociated from the trauma source, remained operative, yet its functional mode appeared to shift from resource buffering in normative contexts to resource amplification under traumatic conditions.

Fourth, work flexibility willingness, family flexibility ability, and family flexibility willingness exhibited no significant moderating effects (Hypothesis 4 supported in terms of the statistical pattern). This pattern of dissociation was consistent with the trauma blocking hypothesis, but it is critical to emphasize that non-significant findings do not constitute direct evidence of blocking. Alternative explanations—including measurement limitations, insufficient variance, or cultural factors—cannot be ruled out. Moreover, because the proposed mechanisms (avoidance, emotional numbing, attachment insecurity) were not directly measured, the trauma blocking hypothesis remains a tentative framework requiring direct empirical testing in future research.

In sum, this investigation provided preliminary empirical findings consistent with the trauma blocking hypothesis, suggesting domain-specific differentiation of resource efficacy under traumatic conditions. The findings extended work–family border theory by indicating trauma as a potential boundary condition, refined conservation of resources theory by highlighting contextual parameters that may govern resource functionality, and offered a novel framework for understanding occupational mental health in traumatized populations. At the practical level, the results suggested that educational administrators might consider prioritizing objective work flexibility arrangements (e.g., flexible scheduling, task autonomy) over simply encouraging bereaved teachers to ‘seek support from family,’ while simultaneously monitoring teachers’ burnout and existential anxiety levels to facilitate meaning-centered interventions that support post-bereavement meaning reconstruction. However, these practical recommendations should be considered preliminary pending replication and direct testing of the hypothesized mechanisms.

## Data Availability

The datasets presented in this article are not readily available because they contain sensitive information from a bereaved population and participant confidentiality must be protected. Requests to access the datasets should be directed to the corresponding author.
